# PDBe: Protein Data Bank in Europe

**DOI:** 10.1093/nar/gkt1180

**Published:** 2013-11-27

**Authors:** Aleksandras Gutmanas, Younes Alhroub, Gary M. Battle, John M. Berrisford, Estelle Bochet, Matthew J. Conroy, Jose M. Dana, Manuel A. Fernandez Montecelo, Glen van Ginkel, Swanand P. Gore, Pauline Haslam, Rowan Hatherley, Pieter M.S. Hendrickx, Miriam Hirshberg, Ingvar Lagerstedt, Saqib Mir, Abhik Mukhopadhyay, Thomas J. Oldfield, Ardan Patwardhan, Luana Rinaldi, Gaurav Sahni, Eduardo Sanz-García, Sanchayita Sen, Robert A. Slowley, Sameer Velankar, Michael E. Wainwright, Gerard J. Kleywegt

**Affiliations:** Protein Data Bank in Europe, European Molecular Biology Laboratory, European Bioinformatics Institute (EMBL-EBI), Wellcome Trust Genome Campus, Hinxton, Cambridge CB10 1SD, UK

## Abstract

The Protein Data Bank in Europe (pdbe.org) is a founding member of the Worldwide PDB consortium (wwPDB; wwpdb.org) and as such is actively engaged in the deposition, annotation, remediation and dissemination of macromolecular structure data through the single global archive for such data, the PDB. Similarly, PDBe is a member of the EMDataBank organisation (emdatabank.org), which manages the EMDB archive for electron microscopy data. PDBe also develops tools that help the biomedical science community to make effective use of the data in the PDB and EMDB for their research. Here we describe new or improved services, including updated SIFTS mappings to other bioinformatics resources, a new browser for the PDB archive based on Gene Ontology (GO) annotation, updates to the analysis of Nuclear Magnetic Resonance-derived structures, redesigned search and browse interfaces, and new or updated visualisation and validation tools for EMDB entries.

## INTRODUCTION

For over 4 decades, scientists working in structural biology and related disciplines have been benefiting from a single global archive of 3D structures of biological macromolecules—the Protein Data Bank (PDB) (1–3). Since 2003, the PDB has been jointly managed by the members of the Worldwide PDB consortium (4–6): the Research Collaboratory for Structural Bioinformatics (RCSB PDB) (7), the Protein Data Bank Japan (PDBj) (8) and the Protein Data Bank in Europe (9–11). In 2006, the Biological Magnetic Resonance Bank (BMRB) (12) joined the wwPDB organization (13). Together, the four partners act as deposition and annotation sites for 3D structures and associated experimental data. They also jointly distribute and remediate the PDB archive, and engage with the wider scientific community on issues of formats, policy and validation criteria. Simultaneously, the wwPDB partners are engaged in friendly competition to develop tools for presenting structural data to their users. PDBe’s motto is ‘Bringing structure to biology’, i.e*.* to make the complex and rich structural and functional information in the PDB more accessible and useful to the biomedical community (9–11,14,15).

In 2002, PDBe pioneered the archiving of electron microscopy (3DEM) volume maps by launching the Electron Microscopy Data Bank (EMDB) (16). Since 2007, the EMDB archive has been jointly managed by PDBe, RCSB PDB and the National Center for Macromolecular Imaging (NCMI) at Baylor College of Medicine, Texas, USA, under the aegis of the EMDataBank organization (17). The relationship between EMDB and EMDataBank is analogous to that between the PDB archive and the wwPDB organization, which manages it.

In this article, we describe updates to the resources, services and tools provided by PDBe since our last publication (9).

## SIFTS: STRUCTURE INTEGRATION WITH FUNCTION, TAXONOMY AND SEQUENCE

The SIFTS resource (http://pdbe.org/sifts) (18,19) provides up-to-date cross-reference information between protein structures (i.e. in PDB entries) and other bioinformatics resources. In the past 2 years, Gene Ontology (GO) (20) and InterPro (21) assignments have been improved substantially. These mappings now apply directly to the protein sequences in PDB entries. Previously, such mappings could pertain to domains that belonged to the mapped UniProt (22) entry, but were not actually present in the protein sample used to determine the structure. Other improvements include the handling of chimeric constructs, microheterogeneity, sequence conflicts between the natural protein and the construct used in the structural study and more up-to-date information on enzymes. It is now also possible to download SIFTS information (in XML format) for proteins in PDB entries with no UniProt mapping. Improvements in the SIFTS infrastructure have resulted in more accurate representation of sequence and cross-reference information on the PDB entry pages of the PDBe website (9–11). As SIFTS mappings are used by a number of large bioinformatics resources worldwide, the information they use and display has also benefited from the improvements.

## GO BROWSER

**Figure 1.**
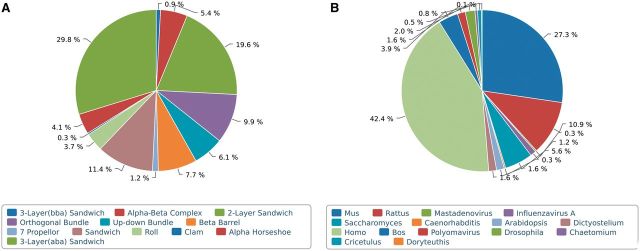
Examples of statistics available from the GO browser module of PDBeXplore (http://pdbe.org/go) for protein structures in PDB entries that have been annotated with the GO term for vesicle-mediated transport (GO identifier 0016192) or any of its children. (**A**) Domain architectures (CATH) (26) observed in the entries annotated with the above GO terms. Just over 50% of the observed proteins have either a 2- or 3-layer sandwich architecture. (**B**) Genera to which the source organisms of the selected proteins belong. About 80% of the studied proteins are from human, mouse or rat.

PDBeXplore (10) is a PDBe service comprising a set of browsers that allow users to explore and analyse the available structure data in the PDB archive for subsets of entries. The user can select subsets of interest based on well-known chemical and biological classification systems such as enzyme classification (EC numbers) (23), NCBI taxonomy database (24), Pfam sequence family data (25) and CATH structure domain classification (26). We have added a new browser module based on GO assignments (20) (http://pdbe.org/go; Figure 1) (15,18). This browser allows the user to find and analyse relevant structures in the PDB based on the three GO categories:

- Molecular function terms, which describe functional activities at the molecular level (e.g. catalytic activity, binding activities);
- Cellular component terms, which describe the cellular location of biomacromolecules (e.g. outer membrane-bounded periplasmic space); and
- Biological process terms, which describe operations or sets of molecular events carried out by macromolecules (e.g. vesicle-mediated transport).

Similarly to other PDBeXplore modules, the GO browser provides a facility to search for a particular GO term, or the user can browse a tree-like representation of the terms. On selection of a GO term, the browser presents the set of PDB entries matching that term via a number of views, designed to answer questions such as (i) Which small molecules are found in the PDB entries matching the GO term? (ii) Which CATH (26) folds are observed (Figure 1A)? (iii) What types of quaternary structures as determined by PISA (27) are found? (iv) Which Pfam (25) sequence families are present? (v) Which taxa do the source organisms of these proteins belong to (Figure 1B)? and (vi) Who has determined these structures and where were they published?

PDBeXplore is a prime example of how the information provided through SIFTS can be used to develop entirely new ways of accessing and analysing the contents of the structure archive.

## NEW BIOLOGY IN THE PDB

Improvements to the SIFTS infrastructure (see earlier in text) now allow the identification of newly released PDB entries that map to a Pfam sequence family, a GO term or a UniProt entry that has not previously been encountered in the PDB. Equally useful is the identification of PDB entries that map to a newly defined Pfam family, GO term, UniProt entry or CATH or SCOP (28) fold, as and when these resources are updated and processed by SIFTS. This information is updated for every weekly PDB release and is presented on the PDBe website (http://pdbe.org/latestbiology), alongside the newly released or updated PDB and EMDB entries (http://pdbe.org/latest) and new or modified small molecules and non-standard polymer residues (http://pdbe.org/latestcompounds).

## BIOBAR

Biobar (11) is a Firefox-compatible toolbar that provides access to a wide range of bioinformatics resources directly in the web browser. Users can query and retrieve data from >45 major resources covering literature as well as genomic, proteomic, functional, taxonomic, structure, plant- and animal-specific databases. Biobar also allows users to highlight any term on a web page and to perform a search for the highlighted keyword. The toolbar further provides information and links to major deposition sites for biomedical data. A new version of Biobar was released in March 2013. It includes access to two additional databases: COSMIC (http://cancer.sanger.ac.uk/cancergenome/projects/cosmic/) (29) and HGNC (http://www.genenames.org/) (30), which provide information on somatic mutations and standard names for human genes, respectively. Moreover, the layout and listing of the databases and links within the toolbar were streamlined and the performance was improved. The latest version of the toolbar is available from https://addons.mozilla.org/en-us/firefox/addon/biobar/.

## 3DEM-RELATED RESOURCES

The EMDB archive (16,17) has more than doubled in size between 2010 and 2013 and now holds over 2000 entries. This growth reflects the increasing popularity of 3DEM as a valuable structural technique. Below is a brief summary of recent updates to 3DEM-related tools and resources at PDBe (http://pdbe.org/emdb), developed largely in response to feedback from the community.

### EMDB statistics

EMStats (http://pdbe.org/emstats) (9) is a service that presents up-to-date EMDB-related statistics as interactive charts. Recent additions include statistics on sample taxonomy, and software and microscope usage. The latter two are calculated either on the basis of the EMDB entries or of the unique publications describing them, thus accounting for the fact that a 3DEM study increasingly involves deposition of multiple volume maps for different states of the macromolecules (e.g. apo- and holo- forms) or of series of subtomogram averages. Other charts show the EMDB entry download trends at all wwPDB partner sites and highlight the top 10 downloaded entries. EMDB deposition and annotation statistics for the wwPDB partner sites are also available.

### Search and navigation of EMDB

EMSearch (http://pdbe.org/emsearch) is a new service for advanced form-based searches of EMDB data. It is powered by a SOLR-based (http://lucene.apache.org/solr/) search engine, benefiting from many standard features, such as handling of ‘*’-wildcards in any position of the search query, truncation and filtering and faceting of the results. EMSearch also supports phonetic searches for author names, as these are often misspelt in queries. EMBrowse (http://pdbe.org/embrowse) is an alternative way to explore the EMDB archive using categories such as EM method (single-particle, tomography and so forth), resolution and source organism of the macromolecular system. It uses the same search engine as EMSearch, thus permitting the user to quickly narrow the search space through the use of filters. The main 3DEM resources page at PDBe (http://pdbe.org/emdb) provides quick-access links to popular search categories (e.g. ribosomes or viruses).

### 3DEM visualization and validation tools

**Figure 2.**
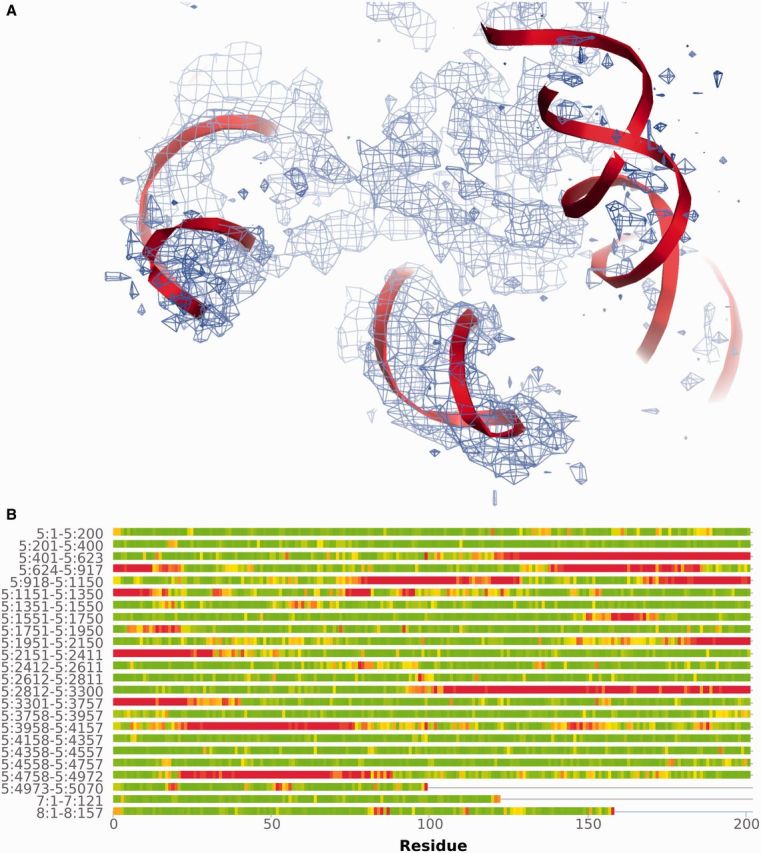
Salient features of the 3DEM volume viewer (**A**) and visual analysis pages (**B**) for human 80S ribosome, EMDB entry EMD-5592 (35) and fitted human 60S rRNA, PDB entry 3J3F (35). (A) Overlay of RNA model (chain 5) and 3DEM map showing regions of good fit (RNA structures on the bottom and left corresponding to nucleotides 2558–2760 and 4129–4157, respectively) and bad fit (RNA structure on the right corresponding to nucleotides 2940–3247). Correspondingly, in (**B**) we can see that nucleotides in the good fit regions are mainly green, whereas those in the bad fit region are mainly red. (B) Atom-inclusion graph for every residue in PDB entry 3J3F at the recommended contour level for EMDB entry EMD-5592. The colour of a residue denotes which fraction of its atoms fits the 3DEM map at the given contour level, varying from red (0%) to green (100%). The chain names in PDB entry 3J3F are numeric (5, 7 and 8).

PDBe offers several methods for the visualization, analysis and validation of 3DEM volume maps, tomograms and fitted models (31). Interactive visualization of volume maps and fitted models is provided via the 3D Volume Viewer browser applet (e.g. http://pdbe.org/emd-1948/volume) (Figure 2A), which is based on the OpenAstexViewer (32,33). The relatively low signal-to-noise ratio in tomograms makes surface rendering of tomographic reconstructions difficult to interpret. Therefore, tomograms in EMDB can be inspected as a set of 2D slices using the OMERO (34) tomogram slice viewer (e.g. http://pdbe.org/emd-2432/slice).

Basic ‘sanity checks’ related to the volume map and the fit of atomic models from the PDB are provided on the Visual Analysis pages at PDBe (Figure 2B) (e.g. http://pdbe.org/emd-5592/analysis). These pages show orthogonal projections of the map and map/model or map/mask overlays for any fitted models or masks (also called segmentations), as well as a number of charts. Often, 3DEM volume maps are manipulated before deposition to filter noise (e.g. zero filling). Such manipulations manifest themselves in a density histogram as a peak at zero. Depositors recommend a contour level for volume maps and are also requested to provide the molecular weight of the macromolecular system, which can be used to estimate its volume. Ideally, the volume estimate from the molecular weight and the volume enclosed by the recommended contour level should be similar. Whether or not this is the case can easily be assessed from a volume versus contour-level plot. The goodness-of-fit between a fitted model and a volume map can be checked with atom-inclusion charts available from the Visual Analysis pages. One kind of graph provides an overall view, showing the fraction of all atoms encompassed by the volume as a function of contour level. An additional plot gives a detailed per-residue analysis of atom inclusion at the recommended contour level (Figure 2B). These charts can be used in combination with the 3D Volume Viewer to assess the correspondence and quality of fit between the model and the map (Figure 2A).

## NMR-RELATED RESOURCES

**Figure 3.**
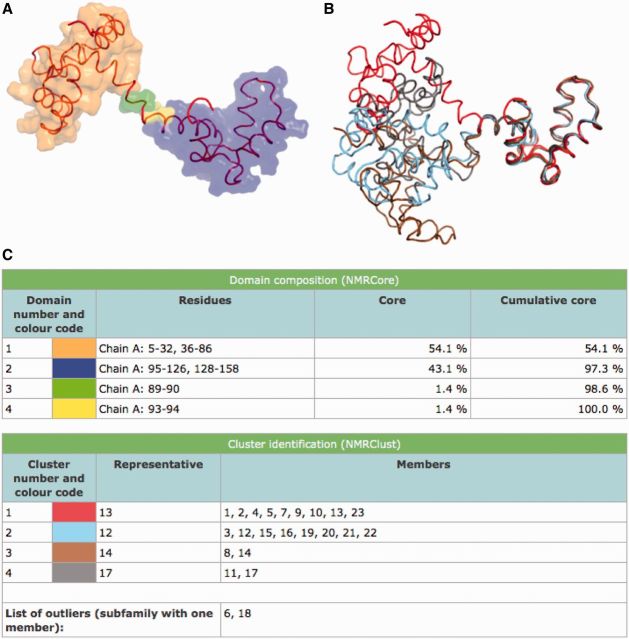
OLDERADO (38) analysis of the NMR ensemble for a skeletal troponin C, PDB entry 1TNW (39). (**A**) Rigid-body domains identified by OLDERADO. The two largest domains (orange and blue) form the two lobes of the protein, whereas the two smaller ones (green and yellow) cover the linker region. (**B**) OLDERADO identified four clusters in the ensemble, and the representative model of each cluster is shown here. (**C**) Summary table of the OLDERADO results. Domains and representative models are colour-coded in exactly the same way as in panels A and B, which facilitates the interpretation of the results.

PDBe provides a variety of NMR-related resources (http://pdbe.org/nmr) (9–11), such as Vivaldi (http://pdbe.org/vivaldi) (36), which uses a number of internal and external data sources to collate and interactively present validation information about NMR structures in the PDB and associated experimental data (chemical shifts and geometric restraints). In addition, the validation of chemical shifts through atomic coordinates (37) is now presented in a more easily accessible manner (http://www.ebi.ac.uk/pdbe/nmr/vasco/), and the presentation of the domain analysis and clustering of NMR ensembles (OLDERADO) (38) has also been improved (http://pdbe.org/olderado). OLDERADO analysis pages include two images (Figure 3) highlighting the rigid-body domains and the representative models from each cluster identified in the NMR ensemble.

## OUTREACH ACTIVITIES

PDBe delivers training at institutions across Europe, both independently and as part of EMBL-EBI’s extensive programme of courses and workshops. These events provide an invaluable opportunity to engage with users and to obtain feedback on the information, tools and services that PDBe provides. A major outreach challenge is making structural knowledge accessible to an increasingly large and diverse audience of non-specialist users (14,15). To help address this challenge, PDBe produces ‘Quips’ articles (9) that allow for interactive analysis and exploration of interesting structures from the PDB archive. Quips articles are often written in collaboration with members of the structural biology community and serve as an ideal starting point for students, teachers and other non-specialist users of structural information to explore and better understand the principles and intricacies of macromolecular structures and how they relate to sequence and function. Social media, including Twitter (http://twitter.com/PDBeurope) and Facebook (http://www.facebook.com/proteindatabank), are increasingly used to engage with the growing online communities and to introduce new users to the fascinating world of structures. YouTube will be used to provide short training videos and other educational material (http://www.youtube.com/user/ProteinDataBank).

## FUNDING

European Molecular Biology Laboratory (EMBL); Wellcome Trust [088944]; European Union [FP7 BioMedBridges 284209]; UK Biotechnology and Biological Sciences Research Council [BB/J007471/1, BB/I02576X/1 and BB/K016970/1]; and the National Institutes of Health, NIGMS [1RO1 GM079429-01A1]. Funding for open access charge: The Wellcome Trust.

*Conflict of interest statement.* None declared.
